# Novel Healthy Eating Index to Examine Daily Food Guides Adherence and Frailty in Older Taiwanese

**DOI:** 10.3390/nu13124210

**Published:** 2021-11-24

**Authors:** Kian-Yuan Lim, I-Chen Chen, Yun-Chun Chan, In-Fai Cheong, Yi-Yen Wang, Zi-Rong Jian, Shyh-Dye Lee, Chi-Chun Chou, Feili Lo Yang

**Affiliations:** 1 Ph.D. Program in Nutrition and Food Science, College of Human Ecology, Fu Jen Catholic University, New Taipei City 242062, Taiwan; limkianyuan@gmail.com; 2 Department of Nutritional Science, College of Human Ecology, Fu Jen Catholic University, New Taipei City 242062, Taiwan; believe850617@gmail.com (I.-C.C.); jenny850823@gmail.com (Y.-C.C.); infaiinfai@gmail.com (I.-F.C.); bebeeateat1005@gmail.com (Y.-Y.W.); qmio137@gmail.com (Z.-R.J.); 3 Department of Family Medicine, Fu Jen Catholic University Hospital, New Taipei City 243089, Taiwan; shyhdye@gmail.com; 4 Department of Otorhinolaryngology, Yonghe Cardinal Tien Hospital, New Taipei City 234408, Taiwan; cthyh10680@gmail.com

**Keywords:** dietary adequacy, dietary quality, nutrient-dense, oldest-old, whole grains

## Abstract

This study was conducted to investigate the adherence of Daily Food Guides (DFGs) among older Taiwanese, and the relationship of dietary quality and frailty. 154 functional independent older adults who were retirement home residents or community dwellers involved in congregate meal services were recruited. DFGs adherence was measured using a novel Taiwanese Healthy Index (T-HEI). Dietary quality was further assessed using Dietary Approach to Stop Hypertension (DASH) and Mediterranean Diet Score (MDS). Frailty was defined using modified Fried’s criteria. Of the total participants, 12.3% were considered non-frail individuals, while 77.3% were prefrail, and 10.4% were frail. Compared to non-frail participants, prefrail and frail individuals indicated significantly lower adherence to DFGs (*p*_trend_ = 0.025). Intake of dark or orange vegetables (*p*_trend_ = 0.010), whole grains (*p*_trend_ = 0.007), as well as nuts and seeds (*p*_trend_ = 0.029) by non-frail individuals were significantly higher than the levels by prefrail and frail individuals. Linear regression model adjusted for age, gender, and functional ability showed that T-HEI was inversely associated with frailty status (β = −0.16 ± 0, *p* = 0.047), but additional adjustment for nutritional status attenuated the association (β = −0.14 ± 0, *p* = 0.103). A similar relationship was observed for DASH but not MDS (DASH: β = −0.18 ± 0.01, *p* = 0.024; MDS: β = −0.06 ± 0.02, *p* = 0.465). After adjustment for confounders, the association was not observed. However, the distribution of whole grains component in both DASH and MDS was significantly higher in non-frail than prefrail and frail individuals, indicating the importance of whole grains intake in frailty prevention. In conclusion, higher adherence to DFGs and better dietary quality were associated with a lower prevalence of frailty. Higher nutrient-dense foods intake such as whole grains, dark or orange vegetables, nuts, and seeds mark a watershed in frailty prevention.

## 1. Introduction

People live longer nowadays; that allows us the opportunity to study in depth the health demand in the later stage of life. Adequate intake of nutritious food for fulfilling the energy and nutritional needs is not only central to health but also determinant to the prevention of age-associated diseases and degeneration [1]. However, along with aging, the change in energy requirement and food consumption may compromise nutritional status thus functional ability among older adults [2]. Age-associated frailty, for instance, is a medical syndrome characterized by decreased physiological function in both strength and endurance which places older adults further on the vulnerability of developing adverse health-related outcomes and dependency [3].

Nutrition has been identified as a vital cornerstone of frailty prevention [4]. In Taiwan, food guidance provided as the Daily Food Guides (DFGs), is an endorsed official document designated to provide culturally appropriate advice and recommendations to bring about a healthy diet in light of nutrient sufficiency and disease prevention across the life cycle [5,6]. However, the population adherence to DFGs has not been investigated since the updated DFGs were released in 2018. Besides, evidence regarding the association between DFGs adherence and frailty in older adults is scarce.

This study aimed to address the current knowledge gap by developing a fresh dietary quality appraisal approach to assess individuals’ adherence to Taiwan DFGs. Furthermore, the compliance level with the DFGs of non-frail older adults’ dietary habits were assessed. Dietary quality was also measured by the Dietary Approach to Stop Hypertension (DASH) score and Mediterranean Diet Score (MDS) because promising results showed that higher adherence was associated with a lower frailty rate [7,8,9]. It is hypothesized that higher adherence to DFGs and better dietary quality are associated with a lower prevalence of frailty.

## 2. Materials and Methods

### 2.1. Study Design and Sampling

Cross-sectional design with older adults aged 65 and above were recruited. Participants who were retirement home residents or community dwellers participating in congregate meal services—a local government-funded program provides meals, mostly lunch, to promote nutritional health and social participation among older adults, in northern Taiwan—were invited if they were free from disability, acute or end-stage diseases, and not in ongoing cancer or disease treatments. Data on sociodemographic, nutritional and dietary status, and physical fitness, were collected by well-trained research assistants. After excluding participants with disabilities in the activity of daily living (*n* = 2) and incomplete data (*n* = 5), 154 cases, provided with written informed consent, participants were enrolled between April 2018 and November 2019. The study protocol was approved by the institutional review board of Fu Jen Catholic University (IRB certificate number: C106019).

### 2.2. The Frailty Assessment

The frailty was characterized based on modified Fried’s frailty criteria [10]. Individuals without disturbance in shrinking, exhaustion, low physical activity, weakness, and slowness were defined as non-frail. Individuals with 1 to 2 disturbances were defined as prefrail, while disturbances of 3 and above were defined as frail. Details of the assessment are provided in Appendix A.

### 2.3. Dietary Assessment

Dietary data were collected through a food frequency questionnaire (FFQ) or dietary record. For participants in retirement homes, where meals for residents were prepared by catering services seven days a week, five single-day dietary records within a month were randomly collected. For community-dwellers, dietary information was obtained through a face-to-face interview by using a self-report food frequency questionnaire (FFQ) modified from Nutrition and Health Survey in Taiwan (NAHSIT). The development and validation of the original FFQ had been reported elsewhere [11,12,13].

### 2.4. Dietary Quality

Daily food intake based on the FFQ or the average of 5 single-day dietary records was used to calculate the individuals’ dietary quality including Taiwanese Healthy Eating Index (T-HEI), DASH score, and the MDS.

The novel T-HEI was developed based on the identical approach used to construct the Healthy Eating Index of American [14] but modified in line with Taiwanese DFGs. The T-HEI was a 100-point scale to measure daily dietary guidance compliance, which was divided into two parts: the adequacy components for ensuring the adequacy of nutrient intake, and the moderation components for limiting consumption to promote optimal health and chronic disease prevention [15]. For the adequacy components, the least restrictive amount of food intake per 1000 kilocalories (kcal) across the energy level was used as the cutoff point for the maximum score (Supplementary Materials Table S1). Consumption at the level of standard or better was assigned a maximum score, whereas no intake was assigned a minimum score of zero. Scores for intake between none and the maximum standard were calculated proportionally. For the moderation components, the reverse scoring method was used (Table 1). The details on the scoring of T-HEI are described in Appendix B.

The original DASH score was a 40-point scale focused on 8 dietary components related to hypertension prevention: high intake of fruits, vegetables, nuts and legumes, whole grains, low-fat dairy, and low intake of sodium, red and processed meat, and sweetened beverage [16]. Individuals’ intake was classified into quintiles. For the adequacy components, quintile 1 was assigned 1 point while quintile 5 was assigned 5 points. For the less healthy components, reverse scoring was applied (Supplementary Materials Table S2). Two adjustments were made in the DASH score to compensate for scoring limitations due to lower intake of low-fat dairy and sweetened beverages. The low-fat dairy component was replaced by dairy and the intake of sweetened beverages was classified into tertiles, with tertile 1 scoring 1 point, tertile 2, 2.5 points, and tertile 3, 5 points.

The MDS was a 9-point scale demonstrating the extent of adherence to the traditional Mediterranean diet [17,18]. For the beneficial components including vegetables, legumes, fruits and nuts, whole grains and cereals, and fish, consumption above the sex-specific median cutoff was assigned 1 point whereas consumption below cutoff assigned a zero. For ethanol, a score of 1 point was assigned to men and women who were moderate drinkers (10–50 g/day for men or 5–25 g/day for women). For lipid intake, the ratio of monounsaturated fatty acids to saturated fatty acids (SFAs) was used. A value above the sex-specific median was assigned 1 point while a value below the sex-specific median was assigned a score of zero. For detrimental components, like dairy, meat and meat products, reverse scoring was applied (Supplementary Materials Table S3).

### 2.5. Statistical Analysis

Statistical power analysis was performed by using G-power 3.1 (Franz Faul, Universitat Kiel, Kiel, Germany). The effect size was 0.05 determined by using T-HEI as the predictor to frailty with a correlation coefficient of −0.221. With a total sample size of 154, post-hoc statistical power of 0.79 was achieved. Characteristics of participants were reported for the overall participants, and by status of frailty as well. Mean and standard deviations (SD) or frequency and percentage were reported for sociodemographic characteristics of participants. Dietary data and dietary quality score distribution were reported as median and interquartile range (IQR). The Jonckheere-Terpstra test was used for comparison across frailty status. The association between dietary quality and frailty was examined with linear regression analyses. Models were further adjusted for potential confounders including age, gender, functional ability, and nutritional status. Analyses were performed using IBM SPSS statistic version 24 (IBM SPSS Statistics, Armonk, NY, USA: IBM Corporation).

## 3. Results

The total participants aged 77.1 ± 7.4 on average overall with the majority of them being female (67.5%) and community dwellers (62.3%). According to the frailty phenotype criteria, non-frail older adults constitute 12.3% of the total participants. A total of 77.3% of participants were identified as prefrail, while 10.4% were frail. Slowness was identified as the major symptom in both prefrail and frail individuals. When compared to the non-frail individuals, prefrail and frail individuals were significantly older (Table 2).

Table 3 shows the median (IQR) of dietary quality score distribution of overall participants as well as by frailty status, while calorie intake and intake of six food groups per 1000 kcal were shown in Table S4. The median (IQR) of the T-HEI score was 61.0 (55.6–71.5) for overall participants. The scores decreased significantly along with worsening frailty status (*p* for trend = 0.025), with median (IQR) of 66.5 (55.2–77.2), 61.3 (55.6–71.8), and 57.6 (51.6–62.7) for non-frail, prefrail, and frail participants, respectively. The component score for ‘nuts and seeds’ was close to zero among the participants, while the component scores for ‘total vegetables’, ‘dark or orange vegetables’, ‘total protein foods’, ‘plant proteins or seafood’, and ‘alcohol’ were toward maximals for the majority participants. Compared with non-frail individuals, prefrail and frail cases had significantly lower scores in ‘dark or orange vegetables’ (*p* for trend = 0.010), ‘whole grains’ (*p* for trend = 0.007), as well as ‘nuts and seeds’ (*p* for trend = 0.029) components. Also, there was a borderline significance trend in the component scores of whole fruits (*p* for trend = 0.072), dairy (*p* for trend = 0.096), and refined grain (*p* for trend = 0.074). DASH score distribution showed borderline significance (*p* for trend = 0.051) among individuals across frailty status. MDS, on the other hand, did not vary across frailty status (*p* for trend = 0.488). The scores of whole grains component in both DASH and MDS were significantly higher in non-frail individuals in comparison to those in prefrail and frail groups. The correlation analysis of T-HEI, DASH score, and MDS indicated a significant correlation between T-HEI and DASH score (*r* = 0.820, *p* < 0.001), as well as MDS (*r* = 0.161, *p* < 0.05) (results shown in Supplementary Materials Figure S1).

**Figure 1:**
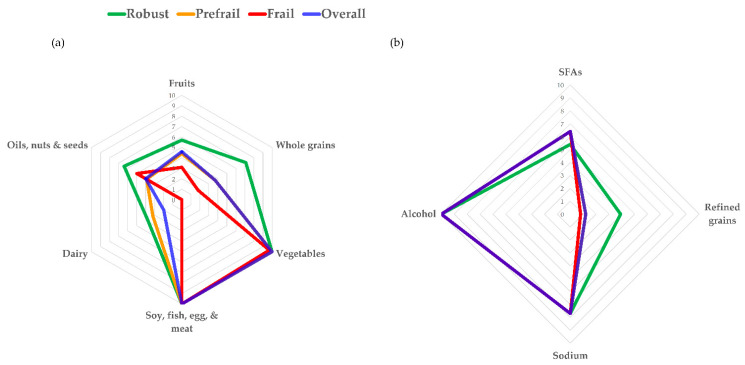
Radar charts for Taiwanese Healthy Eating Index (T-HEI) score distribution for overall participants and across frailty status in (**a**) T-HEI integrated scores, based on six food groups from DFGs, and (**b**) T-HEI moderations components scores.

Figure 1a illustrates T-HEI integrated scores distribution of six food groups for the overall participants and varied frailty status, designated by colors. It is shown that the scoring pattern among prefrail individuals (presented in color orange) was similar to the overall population (presented in color blue) except with a higher dairy component score. The median scores for fruits, whole grains, dairy, and oils, nuts, and seeds of non-frail individuals (presented in color green) were amongst the highest, which was above the overall median (presented in color blue). In contrast to non-frail individuals, the median scores for fruits, whole grains, and dairy of frail cases (presented in color red) were not only amongst the lowest but also below the overall median. The median score for oils, nuts, and seeds, was higher in frail cases than the prefrail cases and overall median, but lower than non-frail cases. All participants had similar median scores in both protein-rich foods and vegetables components scores. Figure 1b illustrates T-HEI moderation components scores distribution for overall participants and based on frailty status. It was found that the median score of prefrail and frail individuals generally overlapped with the overall median. Nevertheless, a lower SFAs component score and higher refined grains component score were observed in non-frail individuals, which indicated a higher intake of SFAs and lower consumption of refined grains.

The association of dietary quality and frailty status is shown in Table 4. The results suggest that higher dietary quality scores of T-HEI and DASH were inversely associated with frailty status, but the association was not observed in the MDS (T-HEI: β = −0.22 ± 0, *p* = 0.006; DASH: β = −0.18 ± 0.01, *p* = 0.024; MDS: β = −0.06 ± 0.02, *p* = 0.465). After adjusting for age, and gender (Model 2), and functional activity (Model 3), the association remained significant only in T-HEI (Model 2: β = −0.19 ± 0, *p* = 0.022; Model 3: β = −0.16 ± 0, *p* = 0.047). Additional adjustment for nutritional status somewhat attenuated the association (Model 4: β = −0.14 ± 0, *p* = 0.103).

## 4. Discussion

To our knowledge, this is the first study that uses the novel T-HEI, a structured dietary quality appraisal system, to measure food intake in accordance with the DFGs of Taiwan with particular emphasis on the importance of quality aspect. The T-HEI components comprise not only the quantitative adequacy of six major food groups but also incorporate subgroups including whole grains, plant protein and seafood, dark-colored vegetables, as well as nuts and seeds, which are considered nutrient-dense and the emphasis of a healthy eating pattern [19]. Apart from adequate intake, the recommendation on limited intake of health detrimental dietary components such as SFAs, sodium, and excessive alcohol are also reflected in the moderation components in the T-HEI. The refined grain is included as a moderation component since the domestic survey revealed concern on overconsumption of staple foods among older adults, especially frail individuals [20].

Our findings suggested that non-frail older Taiwanese were higher adherent to DFGs. A visual discrepancy was observed in the T-HEI integrated score in the portion of six food groups among non-frail, prefrail, and frail individuals. In addition, the T-HEI components scores showed a significant trend of increased consumption of nutrient-dense choices in food subgroups such as whole grain from total grain, dark-colored vegetable from total vegetable, as well as nuts and seeds from oils, in non-frail individuals. This may indicate that apart from meeting quantitative adequacy intake, healthy food choices are also key to frailty prevention. Findings from our study are comparable with the research conducted by Lo and colleagues which suggested higher consumption of whole grains, vegetables, nuts and seeds, and dairy, were beneficial in frailty prevention [21].

Several mechanisms may explain the relationship between nutrition and frailty, with changes in body composition accompanied by age progression being the most apparent factor. Aging is characterized by a decrease in muscle with a concomitant increase in body fat. Progressive loss of both muscle mass and strength, defined as sarcopenia, has been a key in the development of frailty [22]. Impaired muscle function leads to physical limitation and declined activity level, thus decreasing basal metabolic rate and alters energy requirement. The imbalance between energy intake and expenditure directly results in malnutrition in older adults. The impaired micro- and macro-nutrients status, in turn, accelerates the loss of muscle mass. Adequate total protein intake (≥1.2 g/kg body weight) from food sources has been identified among the dietary determinants in frailty prevention [23]. In contrast to other studies, intake of protein-rich foods does not show relevance to frailty in our study [24,25]. A possible explanation is that about one-third of our participants were recruited from retirement homes and the rest from community congregate meal sites. For the retirement homes residents, catered meals were provided to every resident for breakfast, lunch, and dinner. For the congregate meal site participants, lunch was provided. A standard meal for both retirement homes and congregate meal sites consisted of a main dish, two side dishes, a staple, and soup. The main dish was a protein-rich food such as meat or fish, while the side dishes were usually vegetables or soy products like tofu. As for the staple, it was commonly white rice or alternatively, whole grain such as brown and purple rice. Since most of our participants had at least one standard meal daily, it is not surprising that their T-HEI integrated score of protein-rich food is toward maximum and does not vary across frailty status. It is worth mentioning that dairy does show a borderline significant disparity in consumption across frailty status. A recent study indicated that higher dairy product intake reduced the risk of frailty and improved skeletal muscle mass by increasing total protein amount in daily diet [26]. Several studies also suggested total protein intake from a variety of food sources rather than homogeneity was inversely associated with the prevalence of frailty [27,28]. In addition to protein, dairy is rich in calcium, which plays an important role in muscular health, functioning in muscle contraction [29].

Failure in adapting to oxidative stress and inflammatory changes during aging is also responsible for frailty pathogenesis [30,31]. Oxidative stress and inflammaging, a chronic low-grade pro-inflammatory state, contribute to the catabolism of skeletal muscle and adipose tissue, as well as decreases myoblast proliferation, which may lead to muscular damage and weakness [32,33]. It is also associated with anorexia, the culprit of compromised nutritional status and weight loss that characterizes frailty [34]. Nutrients play an essential role in the antioxidative defense system as well as immune function. Minerals such as copper, zinc, manganese, and selenium function as an integrated part of important enzymes in combating oxidative damage [29], while vitamin C and vitamin E are non-enzymatic mechanisms in reducing oxidative stress [35]. The relationship between inflammation and the quality of dietary fat has been well documented. Higher total fatty acids and SFAs intake are believed to be pro-inflammatory, while omega-3 fatty acids consumption exerts an inhibitory effect on the activation of innate and adaptive immunity [36,37]. Phytonutrients like carotenoids and flavonoids, which are abundantly found in deeply pigmented vegetables, have also been gaining interest in their antioxidative properties and contribution to reducing inflammatory response [38,39]. However, calorie-rich does not necessarily translate into nutrient-dense. Healthy food choices of whole grains, dark or orange vegetables, nuts, and seeds rich in vitamins, minerals, omega-3 fatty acids, and phytonutrients, provide antioxidants and anti-inflammatory nutrients are needed in frailty prevention since evidence representing dietary habits with higher total antioxidant capacity and lower inflammatory potential were present among non-frail older adults [24,40].

Studies have shown the association between adherence to DASH as well as the Mediterranean diet, and lower frailty incidence among older men and women [8,9]. Our study found that better dietary quality measured by the DASH score but not MDS is inversely associated with frailty status. We further confirm that nutritional status is a potential modifier in the relationship between dietary quality and frailty regardless of age and functional ability. Since dietary patterns in Asian culture may be incompatible with those in the Western world, dietary habits that contribute to good health may therefore be different in the Asian population. Nevertheless, there is a significant difference observed in the component score of whole grains in both DASH and MDS, which further highlights the importance of whole grains intake in frailty prevention.

Designed under the concept of DASH, the most updated DFGs published in 2018 provide quantitative recommendations about food groups and healthier choices on foods with higher nutrient density to prevent non-communicable diseases and to address nutritional problems observed in the past NAHSIT, such as vitamin E and calcium insufficiency [6,41]. Several studies regarding the adherence of DFGs had been carried out, yet were quantitatively focused. The Dietary Diversity Score (DDS) was derived from food intake information reflecting the consumption of six food groups [42]. The DFGs Index measured the absolute food consumption based on calorie levels [43]. The T-HEI, on the other hand, assesses how well dietary habits align with the DFGs not only in quantitative terms but also qualitatively, which is not achieved in the DDS nor DFGs index. However, our findings were in line with the outcome of the NAHSIT 2013–2016, indicating that older Taiwanese might not be dedicated to DFGs adherence, which may imply a lack of educational campaign on DFGs since 2018. In addition to the proactive promotion, conventional provision of nutrient-dense foods in catering services or congregate meals is advised. In particular, dark or orange vegetables are rich in calcium and riboflavin, can be an alternative to dairy since more than 80% of older Taiwanese did not consider dairy as habitual food [44,45].

Setting cutoff points in the T-HEI components is challenging. Scoring criteria for components with a definite standard is straightforward. Starting with a score of zero for no intake, increase that as intakes increase until meeting the least restrictive standards, and vice versa. However, it is less clear for SFAs and sodium even though standard for the minimum scores is achievable based on Dietary Reference Intakes (DRIs) levels. Thus how low of an intake deserves a perfect score remains a debate. The distribution of 1-day SFAs intake of our entire study participants showed that 15% of intake was below 6% of total energy. As the most recent American Heart Association guidelines suggest to aim for a dietary pattern that achieves 5–6% of calories from SFAs, we set our standard at this level for the maximum SFAs component score [46]. A similar approach to SFAs is used in sodium. We set the cutoff point for the maximum score for sodium at 15% of sodium intake distribution among our study participants based on the guidelines for the management of hypertension established by the Taiwan Society of Cardiology and the Taiwan Hypertension Society, who recommended a salt restriction of 2–4 g per day, equivalent to 800 mg/1000 kcal of sodium [47].

There are strengths and limitations in our study. Limited to the cross-sectional design, the causality between DFGs adherence and frailty cannot be established. Due to the small sample, the generalizability of our findings remains to be affirmed in future studies. Nevertheless, the world is experiencing rapid aging with greater growth in the number of the oldest-old adults, more data are needed to better understand the nutritional demand for the very old. In our study, we recruited socially active older Taiwanese from institutions and communities; of the total participants one-third aged above 80 years old. Findings from our study provide crucial information in not only nutrition in the very old but also dietary strategy for staying functionally independent during the later stage of life. A high proportion of very old adults in our study is a possible reason for higher prefrail prevalence as compared to the other [21]. Furthermore, dietary data collected by self-report FFQ are prone to bias with regard to accurate estimates of food frequency and amount. However, food pictures with standard portion sizes were used in our study to minimize the potential error.

## 5. Conclusions

In conclusion, better adherence to DFGs indicated by a higher T-HEI score is associated with a lower prevalence of frailty. Intake of higher nutrient-dense foods, particularly whole grains, dark or orange vegetables, dairy, nuts, and seeds, marks a watershed in frailty prevention.

## Figures and Tables

**Table 1:** Taiwanese Healthy Eating Index (T-HEI) components and standards for scoring.

Component	Standards for Minimum Score of Zero	Standards for Maximum Score	Maximum Score
Adequacy			
Whole fruits	No whole fruits	≥1.1 serving per 1000 kcal	10
Total vegetables	No vegetables	≥1.7 serving per 1000 kcal	5
Dark or orange vegetables	No dark or orange vegetables	≥0.6 serving per 1000 kcal	5
Whole grains	No whole grains	≥2.0 serving per 1000 kcal	10
Total protein foods	No protein foods	≥2.5 serving per 1000 kcal	5
Plant proteins & seafoods	No plant proteins & seafood	≥0.8 serving per 1000 kcal	5
Dairy	No dairy	≥0.6 serving per 1000 kcal	10
Fatty acids	>40% of total energy (90th percentile of distribution)	20–30% of total energy	5
Nuts & seeds	No nuts & seeds	≥0.4 serving per 1000 kcal	5
Moderation			
Saturated fats	>10% of total energy	≤ 6% of total energy (15th percentile of distribution)	10
Refined grains	>4.4 serving per 1000 kcal	≤1.7 serving per 1000 kcal	10
Sodium	>1150 mg per 1000 kcal	≤800 mg per 1000 kcal (15th percentile of distribution)	10
Alcohol	>20 g/day alcohol for man>10 g/day alcohol for woman	No alcohol	10

**Table 2:** Characteristics of overall participants and across frailty status ^1^.

	Overall (*n* = 154)	Non-Frail (*n* = 19)	Prefrail (*n* = 119)	Frail (*n* = 16)	*p*-Value ^2^
Age, years	77.1 ± 7.4	72.6 ± 5.6 ^a^	77.6 ± 7.3 ^b^	78.6 ± 8.5 ^a,b^	0.015
below 80	97 (63.0)	16 (84.2)	71 (59.7)	10 (62.5)	
80 and above	57 (37.0)	3 (15.8)	48 (40.3)	6 (37.5)	
Accommodation					0.353
Retirement home	58 (37.7)	5 (26.3)	45 (37.8)	8 (50)	
Community	96 (62.3)	14 (73.7)	74 (62.2)	8 (50)	
Gender					0.529
Male	50 (32.5)	5 (26.3)	38 (31.9)	7 (43.8)	
Female	104 (67.5)	14 (73.7)	81 (68.1)	9 (56.3)	
BMI	24.9 ± 3.6	23.9 ± 2.3	24.9 ± 3.6	25.7 ± 2.9	0.242
MNA (0–30)	26.9 ± 2.0	27.6 ± 2.2 ^a^	26.9 ± 2.0 ^a,b^	26.2 ± 1.8 ^b^	0.015
ADL (0–100)	98.2 ± 10.6	98.1 ± 6.9	98.7 ± 10.6	94.7 ± 8.8	0.159
IADL (0–24)	20.4 ± 4.8	21.9 ± 3.7 ^a^	20.7 ± 4.0 ^a^	16.3 ± 6.8 ^b^	0.003
Number of frailty criteria (0–5)	1.5 ± 0.9	0 ^a^	1.5 ± 0.5 ^b^	3.2 ± 0.4 ^c^	<0.001
Exhaustion	26 (16.9)	0	15 (12.6)	11 (68.8)	
Weakness	59 (38.3)	0	44 (37)	15 (93.8)	
Low physical activity	8 (5.2)	0	0	8 (50)	
Shrinking	2 (1.3)	0	1 (0.8)	1 (6.3)	
Slowness	132 (85.7)	0	116 (97.5)	16 (100)	

Abbreviations: ADL, activity of daily living; BMI, body mass index; IADL, instrumental activity of daily living; MNA, mini nutritional assessment, SD, standard deviation. ^1^ Values are means ± SDs or frequency (percentage). ^2^ Based on Kruskal-Wallis test with Bonferroni’s correction for continuous variables; or χ^2^ for categorical variables. ^a, b, c^ Nonindentical supescript indicated significant difference.

**Table 3:** Median (IQR) distribution of dietary quality total and components scores for overall participants and across frailty status ^1^.

	Overall (*n* = 154)		Non-Frail (*n* = 19)		Prefrail (*n* = 119)		Frail (*n* = 16)		*p*_trend_ ^2^
T-HEI	61.0	(55.6–71.5)	66.5	(55.2–77.2)	61.3	(55.6–71.8)	57.6	(51.6–62.7)	0.025
Whole fruits	4.6	(3.1–8.3)	5.7	(3.1–10.0)	4.4	(3.1–8.3)	3.1	(0.1–7.5)	0.072
Total vegetables	5.0	(5.0–5.0)	5.0	(5.0–5.0)	5.0	(5.0–5.0)	5.0	(4.7–5.0)	0.548
Dark or orange vegetables	5.0	(4.7–5.0)	5.0	(5.0–5.0)	5.0	(4.7–5.0)	4.7	(4.7–5.0)	0.010
Whole grains	3.7	(1.8–7.2)	7.1	(2.5–10.0)	3.7	(1.8–7.2)	1.8	(0.7–3.3)	0.007
Total protein foods	5.0	(4.8–5.0)	5.0	(4.1–5.0)	5.0	(4.8–5.0)	5.0	(4.9–5.0)	0.350
Plant proteins or seafoods	5.0	(5.0–5.0)	5.0	(5.0–5.0)	5.0	(5.0–5.0)	5.0	(5.0–5.0)	0.867
Dairy	2.0	(0–8.9)	3.8	(1.4–10.0)	3.2	(0–9.2)	0.0	(0–6.6)	0.096
Fatty acids	4.0	(1.9–5.0)	4.7	(1.4–5.0)	3.9	(2.1–5.0)	5.0	(0.3–5.0)	0.772
Nuts & seeds	0.0	(0–4.4)	1.7	(0–5.0)	0.0	(0–4.4)	0.0	(0–0.9)	0.029
Saturated fatty acids	6.4	(2.2–6.4)	5.4	(0–10.0)	6.4	(2.3–6.4)	6.4	(0.6–6.4)	0.842
Refined grains	1.2	(0–4.9)	3.9	(1.1–7.3)	1.2	(0–4.8)	0.8	(0–4.2)	0.074
Sodium	7.7	(7.6–8.7)	7.7	(0–10.0)	7.7	(7.6–8.6)	7.7	(7.6–9.3)	0.958
Alcohol	10.0	(10.0–10.0)	10.0	(10.0–10.0)	10.0	(10.0–10.0)	10.0	(10.0–10.0)	0.901
DASH	24	(22–29)	27	(20–30)	22	(22–29)	22	(21–26)	0.051
Fruits	3	(2–4)	4	(2–4)	3	(2–4)	2	(1–3)	0.064
Vegetables	3	(2–4)	4	(1–5)	3	(2–4)	3	(2–3)	0.222
Nuts & legumes	3	(2–4)	2	(1–4)	3	(2–4)	4	(2–4)	0.219
Whole grains	3	(1–5)	5	(2–5)	3	(1–5)	1	(1–3)	0.003
Dairy	1	(1–3)	2	(1–5)	2	(1–3)	1	(1–3)	0.186
Sodium	3	(3–4)	3	(1–5)	3	(3–4)	3	(3–4)	0.621
Red & processed meat	3	(2–4)	4	(2–4)	3	(2–4)	3	(2–4)	0.384
Sweetened beverage	5	(5–5)	5	(5–5)	5	(5–5)	5	(5–5)	0.856
MDS	4	(3–6)	5	(3–5)	4	(3–6)	4	(3–5)	0.488
Vegetables	1	(0–1)	1	(0–1)	1	(0–1)	0	(0–1)	0.247
Legumes	1	(0–1)	0	(0–1)	1	(0–1)	1	(0–1)	0.054
Fruits & nuts	1	(0–1)	1	(0–1)	1	(0–1)	1	(0–1)	0.283
Cereals/whole grains	1	(0–1)	1	(0–1)	1	(0–1)	0	(0–0)	0.004
Fish	0	(0–1)	1	(0–1)	0	(0–1)	0	(0–1)	0.593
Ethanol	0	(0–0)	0	(0–0)	0	(0–0)	0	(0–0)	1.000
MUFA: SFA	1	(0–1)	0	(0–1)	1	(0–1)	1	(0–1)	0.377
Dairy	0	(0–1)	0	(0–1)	0	(0–1)	1	(0–1)	0.242
Meat & meat product	0	(0–1)	1	(0–1)	0	(0–1)	0	(0–1)	0.368

Abbreviations: DASH, Dietary Approach to Stop Hypertension; MDS, Mediterranean diet score; MUFA, monounsaturated fatty acid; SFA, saturated fatty acid; T-HEI, Taiwanese Healthy Eating Index; IQR, interquartile range. ^1^ Values expressed as median (IQR). ^2^ Based on Jonckheere-Terpstra test.

**Table 4:** β ± SE of frailty for 1-SD increase in each dietary quality score ^1^.

	T-HEI		DASH		MDS	
	β ± SE	*p*-Value	β ± SE	*p*-Value	β ± SE	*p*-Value
Model 1 ^2^	−0.22 ± 0	0.006	−0.18 ± 0.01	0.024	−0.06 ± 0.02	0.465
Model 2 ^3^	−0.19 ± 0	0.022	−0.14 ± 0.01	0.094	−0.08 ± 0.02	0.341
Model 3 ^4^	−0.16 ± 0	0.047	−0.10 ± 0.01	0.235	−0.09 ± 0.02	0.269
Model 4 ^5^	−0.14 ± 0	0.103	−0.08 ± 0.01	0.349	−0.08 ± 0.02	0.290

Abbreviations: β ± SE, beta coefficients ± standard error; DASH, Dietary Approach to Stop Hypertension; IADL, instrumental activities of daily living; MDS, Mediterranean diet score; MNA, mini nutritional assessment; SD, standard deviation; T-HEI, Taiwanese Healthy Eating Index. ^1^ Frailty as dependent variable. ^2^ Unadjusted. ^3^ Adjusted for age and gender. ^4^ Adjusted for factors in Model 2 and IADL. ^5^ Adjusted for factors in Model 3 and MNA.

## Supplementary Materials

The following are available online at https://www.mdpi.com/article/10.3390/nu13124210/s1, Table S1: Food servings recommendation based on calorie levels and density basis; Table S2: Scoring standards for DASH score and median intake for Q1 (low consumption) and Q5 (high consumption) among participants; Table S3: Median daily consumption for components in MDS based on sex; Table S4: Median (IQR) intake of six food groups per 1000 kcal density for overall participants and across frailty status. Figure S1: Scatter plot for correlation analysis of (a) T-HEI and DASH score, and (b) T-HEI and MDS.

## Appendix A. The Assessment of Frailty

Shrinking was defined as unintentional weight loss greater than 3 kilograms (kg) over the past three months. Exhaustion was defined as a negative response to the item on the Geriatric Depression Scale—Short form (GDS-15): ‘Do you feel full of energy?’ [48] Low physical activity was defined as below moderately leisure activities participation in World Health Organization Quality of Life—BREF (WHOQoL-BREF) instrument, Taiwanese version [49]. Upper extremity muscle strength was assessed through handgrip strength of the dominant hand which was measured by using JAMAR^®^ hydraulic hand dynamometer. Weakness was defined as grip strength below 28 kg in men or below 18 kg in women [22]. For the slowness, participants were asked to complete the 3-m timed-up-and-go test. Gait speed (m/s) was calculated by distance completed (6 m) divided by time spent. Gait speed below 1.0 m/s was defined as slowness [22].

## Appendix B. The Scoring Criteria of T-HEI

In Taiwan, foods are divided into six major groups including grains, fruits, vegetables, dairy, oils including nuts and seeds, and protein-rich foods such as soy, fish, egg, and meat according to types and nutrients contents they contribute to diet. For each food group, there are measured or weighed food substitutes with nearly identical nutritional content within group. For example, 40 g of cooked rice or 20 g of oatmeal is equivalent to one serving of grains which provides 70 kilocalories (kcal) of energy with 2 g of protein and 15 g of carbohydrates. For fruits and vegetables, a serving is equivalent to 100 g of most fruits and vegetables which provides 60 kcal of energy with 15 g of carbohydrate, and 25 kcal of energy with 1 g of protein and 5 g of carbohydrates, respectively. For protein-rich foods such as medium-fat meat, one serving is equivalent to 80 g of tofu, or 35 g of salmon, or 80 g of egg, or 40 g of low-fat bacon, which provides 75 kcal of energy with 7 g of protein and 5 g of fat (Supplementary Materials Table S1).

The T-HEI was a 100-points scale measuring compliance to the dietary guidance that incorporated six major food groups found in DFGs of Taiwan. Additional components like dark or orange vegetables, plant proteins and seafood, saturated fatty acids (SFAs), refined grains, sodium, and alcohol were included in the components of T-HEI because those were the subgroups of six food groups and dietary recommendations suggested in Taiwanese DFGs [15]. The T-HEI was divided into two parts, the adequacy components for ensuring the adequacy of nutrient intake, and the moderation components for limiting consumption to promote optimal health and chronic disease prevention.

In the Taiwanese DFGs, recommendation on food intake was expressed as an absolute amount according to the varied energy levels. To draw a common scoring standard across energy levels, the intake of foods and nutrients in T-HEI were represented on a density basis. For the adequacy components of food groups including whole fruits, total vegetables, dark or orange vegetables, whole grains, total protein foods, plant proteins and seafood, dairy, as well as nuts and seeds, the least restrictive amount of food intake per 1000 kilocalories (kcal) across the energy levels was used as the cutoff point for the maximum score (Supplementary Materials Table S1). Consumption at the standard level or better was assigned a maximum score; while a minimum score of zero was assigned to no intake. Intake between none and the maximum standard was score proportionally. For the oil group, it was expressed as fatty acid components in T-HEI. Consumption between 20% to 30% of total energy based on DRIs was used as the maximum score standard for the component. Fatty acid consumption greater than the 90th percentile of the population distribution, 40% of total energy, was assigned to the minimum score of zero.

For the moderation components, the reverse scoring method was used. A minimum score of zero was assigned to SFAs or sodium consumption beyond DRIs, which were 10% of total energy from SFAs, and 1150 mg per 1000 kcal of sodium, respectively. Intake level below the 15th percentile of population distribution in saturated fat (6% of total energy) or sodium (800 mg per 1000 kcal), a maximum score of 10 was assigned, separately. For the refined grains component, a maximum score of 10 was assigned to the intakes below the least restrictive amount per 1000 kcal across the energy level. The intake level above the most restrictive amount per 1000 kcal across energy level, on the other hand, a minimum score of zero was assigned. As for alcohol consumption, a minimum score of zero was assigned to intake beyond 20 g per day for men or 10 g per day for women. No alcohol consumption was assigned a maximum score of 10. For all the moderation components, score for intake between maximum and minimum standard was determined proportionally.
