# Supplementary material for: Novel Healthy Eating Index to Examine Daily Food Guides Adherence and Frailty in Older Taiwanese
Source: Nutrients. 2021 Nov 24;13(12):4210. doi: 10.3390/nu13124210 (PMC8707448; doi:10.3390/nu13124210)
Supplement: Supplementary file 1 [file nutrients-13-04210-s001.zip › nutrients-1455906-supplementary.pdf]

Supplementary tables

Supplemented Table S1: Food servings recommendation based on calorie levels and density basis [1]

	**Servings based on calorie level (kcal)**							**Servings per 1,000 kcal**						
**Food group**	**1200**	**1500**	**1800**	**2000**	**2200**	**2500**	**2700**	**1200**	**1500**	**1800**	**2000**	**2200**	**2500**	**2700**
Grains (serving) [2]	**6**	**10**	**12**	**12**	**14**	**16**	**16**	**5**	**6.7**	**6.7**	**6**	**6.4**	**6.4**	**5.9**
Whole grains	4	4	4	4	6	6	6	3.3	2.7	2.2	2	2.7	2.4	2.2
Other / refined grains	2	6	8	8	8	10	10	1.7	4	4.4	4	3.6	4	3.7
Beans, fish, egg, & meat (serving) [3]	**3**	**4**	**5**	**6**	**6**	**7**	**8**	**2.5**	**2.7**	**2.8**	**3**	**2.7**	**2.8**	**3**
Plant & seafood proteins	1	1.5	1.5	2	2	2	2.5	0.8	1	0.8	1	0.9	0.8	0.9
Eggs, meat, & poultry	2	2.5	3.5	4	4	5	5.5	1.7	1.7	1.9	2	1.8	2	2
Dairy (serving) [4]	**1.5**	**1.5**	**1.5**	**1.5**	**1.5**	**1.5**	**2**	**1.3**	**1**	**0.8**	**0.8**	**0.7**	**0.6**	**0.7**
Vegetables (serving) [5]	**3**	**3**	**3**	**4**	**4**	**5**	**5**	**2.5**	**2**	**1.7**	**2**	**1.8**	**2**	**1.9**
Dark colored vegetables	1	1	1	1.5	1.5	1.5	1.5	0.8	0.7	0.6	0.8	0.7	0.6	0.6
Other vegetables	2	2	2	2.5	2.5	3.5	3.5	1.7	1.3	1.1	1.3	1.1	1.4	1.3
Fruits (serving) [6]	**2**	**2**	**2**	**3**	**3.5**	**4**	**4**	**1.7**	**1.3**	**1.1**	**1.5**	**1.6**	**1.6**	**1.5**
Oils, nuts & seeds (servings) [7]	**4**	**4**	**5**	**6**	**6**	**7**	**8**	**3.3**	**2.7**	**2.8**	**3**	**2.7**	**2.8**	**3**
Oils (servings)	3	3	4	5	5	6	7	2.5	2	2.2	2.5	2.3	2.4	2.6
Nuts and seeds (serving)	1	1	1	1	1	1	1	0.8	0.7	0.6	0.5	0.5	0.4	0.4

Abbreviation: kcal, kilocalories.

[1] Based on Daily Food Guides Taiwan

[2] Provides 70 kcal with 2 grams of protein, and 15 grams of carbohydrate in each serving.

[3] Provides 55 kcal with 7grams of protein and 3 grams of fat in each serving of lean meat; 75 kcal with 7 grams of protein and 5 grams of fat in each serving of medium fat meat; 120 kcal with 7 grams of protein and 10 grams of fat in each serving of high fat meat.

[4] Provides 150 kcal with 8 grams of protein, 8 grams of fat, 12 grams of carbohydrate in each serving.

[5] Provides 25 kcal with 1 gram of protein and 5 grams of carbohydrate in each serving.

[6] Provides 60 kcal with 15 grams of carbohydrates in each serving.

[7] Provides 45 kcal with 5 grams of fat in each serving.

Supplemented Table S2: Scoring standards for DASH score and median intake for Q1 (low consumption) and Q5 (high consumption) among participants [1]

Component	Scoring criteria	Q1, servings/day	Q5, servings/day
**Adequacy**			
Fruits	Q1 = 1 point<br>Q2 = 2 points<br>Q3 = 3 points<br>Q4 = 4 points<br>Q5 = 5 points	0 (0 – 0.1)	2.3 (2.0 – 3.1)
Vegetables		2 (1.9 – 2.0)	5.9 (4.1 – 6.1)
Nuts & legumes		0.5 (0.2 – 0.7)	5.7 (4.8 – 6.3)
Whole grains		0.3 (0.2 – 0.5)	2.5 (2.2 – 4.2)
Dairy		0 (0 – 0)	1.1 (1.1 – 1.6)
**Reverse scoring**			
Sodium (mg)	Q1 = 5 points<br>Q2 = 4 points<br>Q3 = 3 points<br>Q4 = 2 points<br>Q5 = 1 point	936.5 (604.8 – 1209.5)	3106.4 (2902.9 – 4160.5)
Red & processed meat		0.3 (0.3 – 0.5)	2.2 (2.0 – 3.0)
Sweetened beverage [2]		0 (0 – 0)	0.8 (0.3 – 1.1)

Abbreviations: DASH, Dietary Approach to Stop Hypertension; IQR, interquartile range; Q, quintile.

[1] Values are servings/day, expressed as median (IQR); except for sodium, expressed as mg.
[2] Grouped into tertiles due to only few cases consumed sweetened beverage.

Supplemented Table S3: Median daily consumption for components in MDS based on sex [1]

	Sex-specific median	
**Component**	**Men (n= 50)**	**Women (n= 104)**
**Beneficial components**		
Vegetables	3.86	3.90
Legumes	0.93	1.30
Fruits & nuts	1.27	0.68
Whole grains / cereal	1.17	0.97
Fish	1.21	1.07
Ethanol	≥ 10 g & < 50 g	≥ 5 g & < 25 g
MUFA : SFA	0.72	1.24
**Detrimental component**		
Diary	0.74	0.13
Meat & meat product	2.60	2.26

Abbreviations: MDS, Mediterranean diet score; MUFA, monounsaturated fatty acid; SFA, saturated fatty acid.

[1] Values are servings/day, expressed as median.

Supplemented Table S4: Median (IQR) intake of six food groups per 1,000 kcal density for overall participants and across frailty status [1]

	Overall (n= 154)		Non-frail (n= 19)		Prefrail (n= 119)		Frail (n= 16)		*P* trend [2]
Calorie intake (kcal)	1690.9	(1481.5 - 2010.1)	1711.2	(1469.6 - 1950.3)	1657.7	(1485.5 - 2010.1)	1890.7	(1533 - 2010.1)	0.507
Total grains	5.2	(3.9 - 5.8)	4.9	(3.9 - 6.5)	5.2	(3.9 - 5.8)	4.7	(3.9 - 5.5)	0.366
Whole grains	0.7	(0.4 - 1.4)	1.4	(0.5 - 2.3)	0.7	(0.4 - 1.4)	0.4	(0.1 - 0.7)	**0.006**
Total vegetables	2.0	(1.9 - 2.6)	2.3	(1.9 - 2.7)	1.9	(1.9 - 2.6)	1.9	(1.7 - 2.5)	0.105
Dark-colored vegetables	0.8	(0.6 - 1.1)	1.0	(0.6 - 1.4)	0.8	(0.6 - 1.1)	0.6	(0.6 - 0.8)	**0.010**
Fruits	0.5	(0.3 - 0.9)	0.6	(0.3 - 1.1)	0.5	(0.3 - 0.9)	0.3	(0 - 0.8)	0.084
Dairy	0.1	(0 - 0.5)	0.2	(0.1 - 0.6)	0.2	(0 - 0.6)	0.0	(0 - 0.4)	0.076
Total protein foods	3.1	(2.4 - 3.5)	3.1	(2 - 3.7)	3.1	(2.4 - 3.5)	3.1	(2.5 - 3.5)	0.623
Plant proteins & seafood	1.4	(1.0 - 2.0)	1.1	(1.0 - 2.0)	1.4	(1.0 - 2.0)	1.5	(1.0 - 1.8)	0.723
Oils, nuts & seeds	3.3	(0.4 - 4.1)	3.7	(0.7 – 4.1)	3.3	(0.4 – 4.2)	1.7	(0.4 – 4.2)	0.358
Nut & seeds	0.0	(0 - 0.3)	0.1	(0 - 0.5)	0.0	(0 - 0.3)	0.0	(0 - 0.1)	**0.028**
Energy from fats (%)	32.0	(29.6 - 36.2)	30.7	(27.8 - 37.2)	32.2	(29.6 - 35.7)	29.6	(29.6 - 39.3)	0.960

Abbreviations: IQR, interquartile range.

[1] Values are servings/day unless otherwise indicated. All values are expressed as median (interquartile range).

[2] Based on Jonckheere-Terpstra test.

Supplementary figure

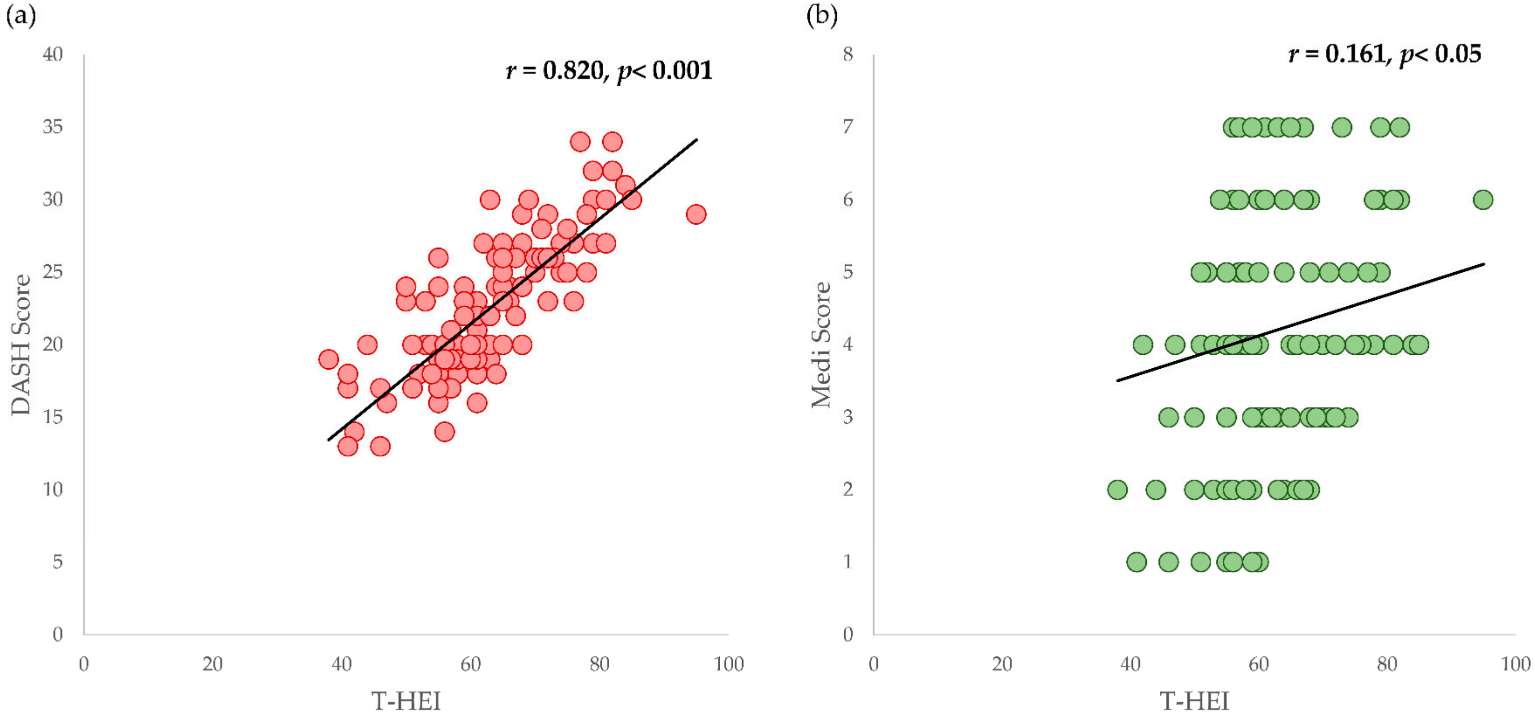

Supplemented Figure S1: Scatter plot for correlation analysis of (a) T-HEI and DASH score, and (b) T-HEI and
